# Changes in Corrosion Behaviour of Zinc and Aluminium Coatings with Increasing Seawater Acidification

**DOI:** 10.3390/ma17030536

**Published:** 2024-01-23

**Authors:** Cezary Senderowski, Wojciech Rejmer, Nataliia Vigilianska, Arkadiusz Jeznach

**Affiliations:** 1 Mechanics and Printing Institute, Faculty of Mechanical and Industrial Engineering, Warsaw University of Technology, Narbutta 85, 02-524 Warsaw, Poland; arkadiusz.jeznach@pw.edu.pl; 2 Department of Materials and Machines Technology, Faculty of Technical Sciences, University of Warmia and Mazury in Olsztyn, 10-719 Olsztyn, Poland; wojciech.rejmer@uwm.edu.pl; 3 Department of Protective Coatings, E.O. Paton Electric Welding Institute, 03680 Kiev, Ukraine; pewinataliya@gmail.com

**Keywords:** arc spraying, protective coatings, corrosion resistance

## Abstract

The increase in greenhouse gas emissions has led to seawater acidification, increasing the corrosion rate of metal structures in marine applications. This paper indicates that the spraying of four types of coatings, namely Zn, Al, Zn-Al, and Al-Mg, using the arc-spraying technique on steel substrate S235JR, creates effective protective coatings that interact differently with various pH solutions exposed to varying levels of seawater acidification. The study analyses the structural properties of the coating materials using SEM and XRD techniques. Electrochemical parameters are evaluated in solutions with different pH and salinity levels. The results demonstrate that alloy metallic coatings provide excellent resistance to corrosion in low-pH solutions.

## 1. Introduction

One of the most relevant problems of the last thirty years is the influence of climate change induced by global warming. This climatic effect is caused by the emission of greenhouse gasses such as CO_2_, CH_4_, and NO_X_ [1]. Greenhouse gases (GHGs) are mainly acidic in character. Therefore, increased greenhouse gas emissions will inevitably lead to reductions in the pH of oceans and seawater. It is estimated that within the next two decades, the pH value of seawater will change from 8 to 7 [2,3,4].

In addition, polluted industrial environments contain acidic ions, such as SO_4_^2−^, CO_3_^2−^ and Cl^−^; these ions originate from industrial effluents and can cause structural failure [5]. It is widely understood that fluid acidity and, in particular, highly aggressive acidic ions are critical factors in the corrosion and degradation of metal alloys [5,6].

Therefore, assessing changes in the service life of coating materials in a changing environment is crucial. Zn, Al, and Mg have anodic characteristics and are primarily used as coating materials to protect steel from corrosion in harsh environments [7,8,9,10,11,12]. Generally, zinc coatings show no significant changes in terms of their corrosion resistance in acidic and alkaline solutions (pH = 4–10) and, therefore, seem to be a good choice for undersea constructions [13]. However, to enhance the corrosion protection of Zn coatings in high-chloride and -acid environments, various types of Zn coatings with Al and Mg have been developed [7,8,11,12,14,15,16]. For instance, ‘Galfan’ Zn coating, which was developed in 1980, contains around 5%wt. Al and exhibits outstanding resistance to chloride corrosion [7]. Similar effects are observed for aluminium coatings, where a stability of corrosion behaviour is observed in a pH range of 3–10 [17].

While aluminium coatings have a wide range of applications in construction, metallurgy, and mechanical engineering, the formation of α–Al_2_O_3_ on the surface of pure aluminium makes it unable to provide direct cathodic protection in seawater [5]. Zn and Mg are more electronegative than Al and dissolve preferentially to form corrosion products that fill the pores and defects in a coating, resulting in increased corrosion resistance. Therefore, adding zinc and magnesium to aluminium coatings enhances the corrosion resistance of steel substrates in specific environments [5].

Generally, magnesium is added to aluminium to improve strength, weldability, and corrosion resistance [18]. However, arc-sprayed Al coatings with Mg have also been found to act as self-healing elements, improving materials’ resistance to electrochemical corrosion in a solution of NaCl+CaCl_2_+NaHCO_3_ with chloride and carbonate ion participation in distilled water at pH 9. This is due to the passive nature of the corrosion products of Mg_6_Al_2_(OH)_16_CO_3_, formed under an aggressive chloride environment, causing them to fill cavities and create a thin layer of stable oxides. In contrast, Al-Zn coatings only offer barrier protection by depositing less stable corrosion products with poorer adhesion. These products do not protect the porous structures of arc-sprayed Al-Zn coatings [5].

Zinc-based and aluminium coatings are applied through sherardising, plating, electroplating, hot-dip galvanising (batch and strip), and thermal spraying (flame, cold gas, and arc) [13,17,18,19,20,21,22,23,24,25].

The coating processes of batch galvanising and thermal spraying are the only ones applicable to large steel structures. The limitations of batch galvanising are component size and the elemental composition of the baths [26]. The arc spray process provides superior coating properties compared to other thermal spray methods for zinc and aluminium coatings [20,21,22,25]. Arc-sprayed Zn and Al coatings are also widely used due to their high temperature resistance, efficient production processes, lower cost, and anti-corrosion protection [27]. Aluminium alloys are the materials suggested most often, with superior performance in seawater and freshwater environments. Thermal-sprayed aluminium coatings are applied to high-temperature materials and equipment made of steel. Aluminium-coated cast-iron components are often exposed to potentially corrosive environments in water and the atmosphere, including combustion gases at high temperatures (900 °C) [24,25,28,29].

This research aims to assess the applicability of thermal-sprayed aluminium, zinc, Zn-Al, and Al-Mg coatings in seawater applications, considering increasing acidity due to global climate changes caused by GHG emissions and industrial pollutants.

## 2. Materials and Methods

### 2.1. Materials and Reagents

Four types of solid feedstock wire materials with a cross-section diameter of 2 mm were selected to produce arc thermal-spraying protective coatings. Commercially pure aluminium wire, produced by MigWeld (GmbH) Co., Ltd. (Landau a der Isar, Germany), and commercially pure zinc wire from MetcoTM (Poznan, Poland) with 99.99% purity, as well as Metco Zn15Al wt.% and Al5Mg wt.% alloy wires (Poznan, Poland), were used in this study. As a substrate material, a steel S235JR plate of 5 mm thickness was used to deposit the coatings using the EM-14M wire arc spray system (Kyiv, Ukraine). The chemical composition of the S235JR steel is presented in Table 1.

Before arc–wire spraying, abrasive blasting of the surface of the S235JR steel samples was performed to provide a roughness of around 110 microns. The wire–arc spray process was carried out at the National Academy of Sciences in Kyiv, Ukraine (Paton Institute); different spraying parameters (presented in Table 2) were applied, influencing the metallurgical quality of the coatings.

Electrochemical analyses were conducted in two distinct solutions. The chemical makeup of the solutions is outlined in Table 3 [31].

The solutions were produced by dissolving sodium chloride (99.8% purity), magnesium chloride hexahydrate (98% purity), sodium sulphate (98.5% purity), calcium chloride (97% purity), potassium chloride (99% purity), and sodium bicarbonate (99.5% purity), purchased from Chembur (Przemyśl, Poland). The solutions were prepared using distilled water produced with a DE 5 Polna laboratory distiller. The target pH for each experiment was attained by titrating with 0.1 M acetic acid solution using a TitroLine 5000 titration apparatus (Xylem Analytics Germany GmbH, Mainz, Germany). Each set involved five evaluations of every substance.

### 2.2. Structural and Surface Analysis

Structural analyses and determinations of the phase composition, as well as the degree of chemical heterogeneity of the coatings, were carried out with Quanta 3D FEG Dual Beam and Philips XL-30/LaB6 scanning microscopes integrated with DX4i–EDAX X-ray microanalysis, as well as with a Seifert 3003 XRD diffractometer with CoKα radiation (λ = 0.178897 nm). An angular step size of 0.02°/min and a step time of 5 s per point were used.

The porosity of the coatings was assessed by photomicrograph quantitative analysis, carried out with (SEM) a Philips XL30/Lab6, programmed with SIS 3.0^®^ software. The Cavaleri-Hacquerta [32] principle was applied, according to which the level of coating porosity is defined with the planimetric method as the ratio of the sum of pore surfaces to the total surface of the specimen.

A PGM-1C profilometer with a G250BS head was used to measure the surface roughness of the coatings. The measurements were carried out at a length of l = 4 mm with a head movement speed of v = 0.2 mm/s and a static load of 3 mN on the blade.

### 2.3. Electrochemical Analysis

The electrochemical properties of the coatings were investigated using an Atlas 1131 Electrochemical Unit and an impedance analyser (Atlas–Sollich, Gdansk, Poland). First, each sample was cleaned using an organic solvent (Isopropanol, 99% purity, Standard Poland). The arc–wire sprayed samples were then positioned within an electrochemical vessel, with the working electrode (WE) containing the material under investigation. The reference electrode (RE) was a silver chloride electrode (Ag/AgCl) and the counter electrode (CE) was made of platinum. An open circuit measurement was performed at the beginning of each measurement to determine the open circuit potential (E_ocp_) values. Following the stabilisation of the sample, a linear polarisation resistance (LPR) test was conducted to obtain polarisation curves and determine the values of corrosion potential (E_corr_), corrosion current density (I_corr_), and approximated corrosion rate (r_corr_).

The outcome of the LPR test resulted in the acquisition of polarographic curves, as described by Equation (1).

E = f(log/I_pol_/) (1)

where:

E is SCE versus WE potential [V], and /I_pol_/ is the modulus of polarisation current density [A × cm^−2^].

Tafel plots were determined based on function (1) to calculate corrosion potential E_corr_ and corrosion current density I_corr_. It is known that corrosion rates are proportional to corrosion current density [33,34]. Therefore, the corrosion rate was calculated using Equation (2), based on Faraday’s first law.

V = I_corr_ × M × n^−1^ × F^−1^ (2)

where V is the corrosion rate (g × m^−2^ × s^−1^), I_corr_ is the corrosion current density (A × m^−2^), M is the molar mass of the metal (g × mol^−1^), n is the number of electrons exchanged in the dissolution reaction, and F is Faradays constant (96,485 C × mol^−1^).

In order to obtain annual values of corrosion rates, the V value was multiplied by a proportionality factor of t, which represents the number of seconds in a year (31,557,600 s × y^−1^) (3).

r_corr_ = V × t (3)

where r_corr_ is the annual corrosion rate (g × m^−2^ × y^−1^), V is the corrosion rate (g × m^−2^ × s^−1^), and t is a proportionality factor (31,557,600 s × y^−1^).

The molar masses of the zinc and aluminium coatings were determined based on the atomic masses of Zn and Al, which are 65.38 g × mol^−1^ and 26.98 g × mol^−1^, respectively. The numbers of exchanged electrons for zinc and aluminium coatings were two and three, respectively. To determine the molar masses of the mixed coatings, atomic ratios were used. The molar masses of the Zn-Al and Al-Mg coatings were 53.6 g × mol^−1^ and 26.8 g × mol^−1^, respectively. The number of electrons exchanged for the mixed coatings was determined through the electrochemical process of the majority element. This resulted in two electrons for the Zn-Al coating and three for the Al-Mg coating. A linear polarisation resistance (LPR) examination was carried out by reading the current reactions of the specimen in the potential spectrum of <E_ocp_ − 0.1 V; E_ocp_ + 0.1 V>. This examination, chosen for its capacity to generate electrochemical values for minor potential changes while minimising any alterations to the specimen’s structure, was conducted at a scanning rate of 1 mV/s and with an exposed surface area of 0.8 cm^2^. Alongside this, Electrochemical Impedance Spectroscopy (EIS) assessments were performed. The experiments were conducted using a voltage amplitude of 0.01 V within a frequency range from 100 kHz to 100 MHz. The replacement electrical unit was adjusted using AtlasCorr11 PC software.

### 2.4. Statistical Analysis and Design of the Experiment

The layout of Table 4 is a basic matrix for 2^2^ experimental designs, from which regression coefficients were calculated for the electrochemical parameters of each material as a function of two environmental factors, i.e., pH and salinity. Salinity values were chosen as those specific for ocean (30 g/L) and river (7 g/L) waters, and pH values were set to correspond to the current acidity of oceans (pH = 8) and values predicted in about 20 years (pH = 7) [2,3,4]. Tests in one solution were performed five times for each material. The statistical significance of the environmental factors was assessed using a two-tailed Student’s *t*-test for α = 0.05. The t-student and linear regression coefficient values were calculated according to the methodology presented by Gadomska-Gajadur et al. [35]. The absolute value of the linear regression coefficient is divided by the average results variance of all used solutions. If the calculated t value is higher than 2.78, the hypothesis of non-influence factor value on the measured parameter cannot be rejected. The mean values of potential and current density were calculated by averaging data from five polarographic plots. The average values of potentials and current density were calculated for measurement in the same order, starting from a transitional point between anodic and cathodic plots. This point is recognised by the change in the current flow direction, indicated by a switch in the sign of the current density value from positive to negative. For the EIS results, average impedance and phase shift values are calculated for given frequencies.

## 3. Results and Discussion

### 3.1. Structure Analysis

**Figure 1:**
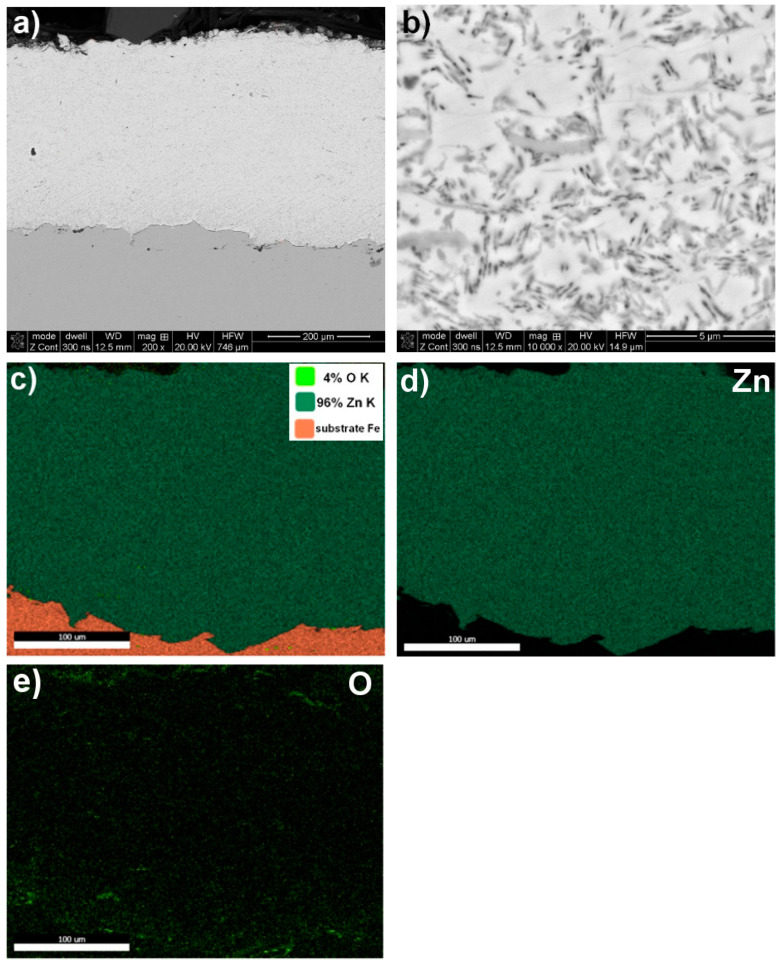
A typical microstructure of Zn arc–wire sprayed coating exhibiting type and grain morphology (**a**,**b**), with chemical composition based on SEM/EDS map analysis (**c**–**e**).

**Figure 2:**
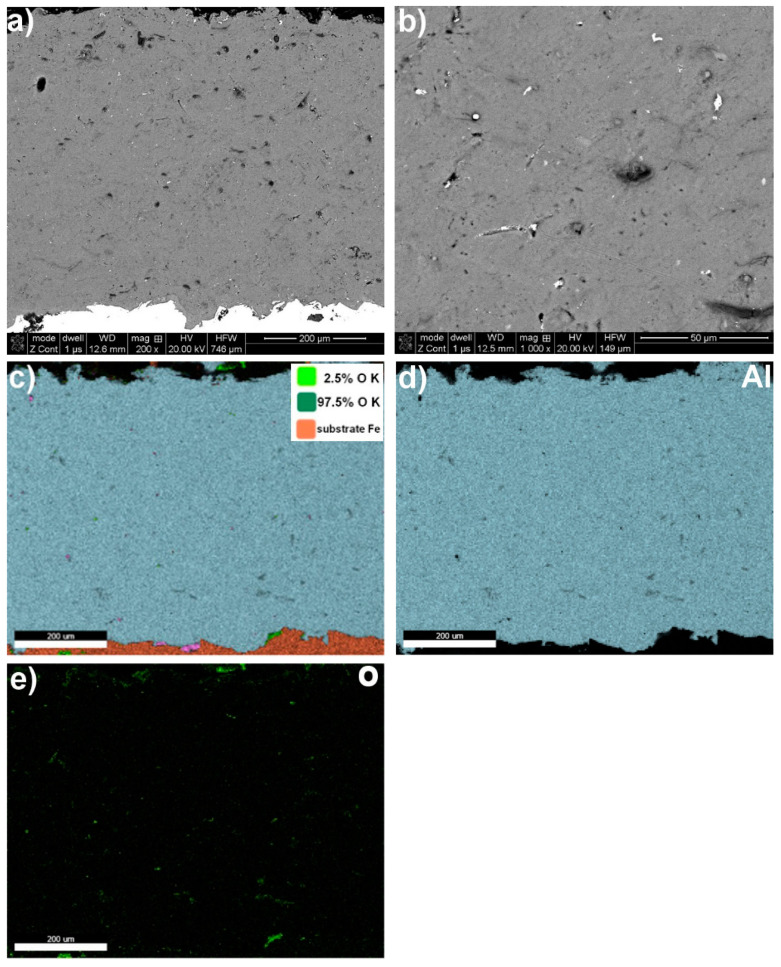
A typical microstructure of an Al arc–wire sprayed coating, exhibiting type and grain morphology (**a**,**b**), with chemical composition based on SEM/EDS map analysis (**c**–**e**).

**Figure 3:**
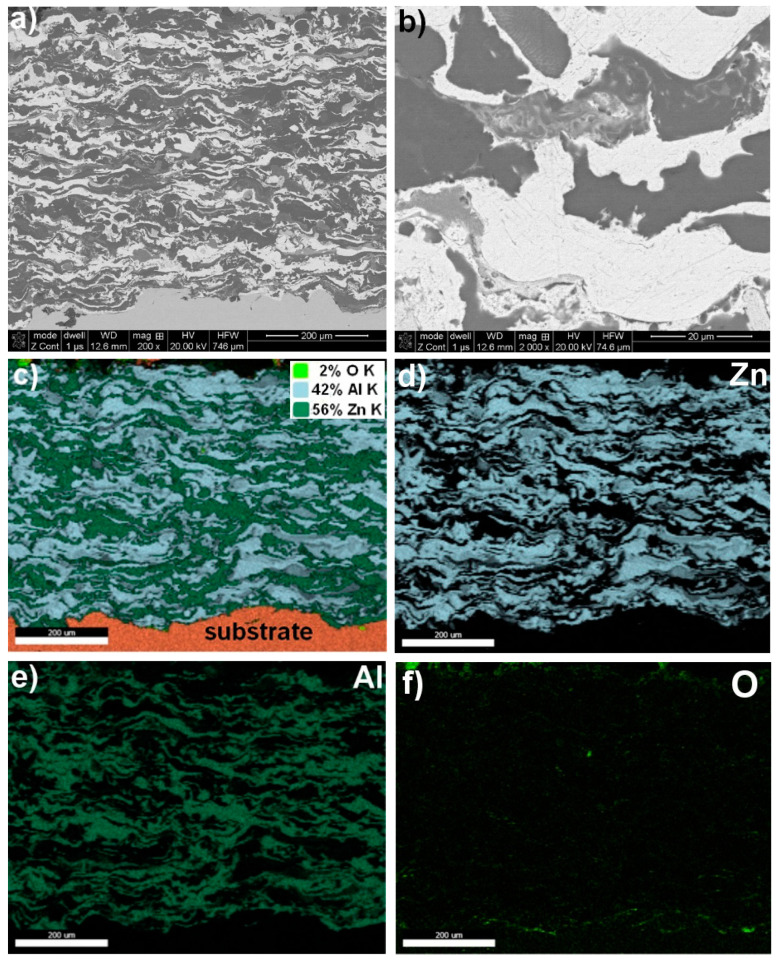
A typical microstructure of Zn15Al arc–wire sprayed coating exhibiting type and grain morphology (**a**,**b**) with chemical composition based on SEM/EDS map analysis (**c**–**f**).

**Figure 4:**
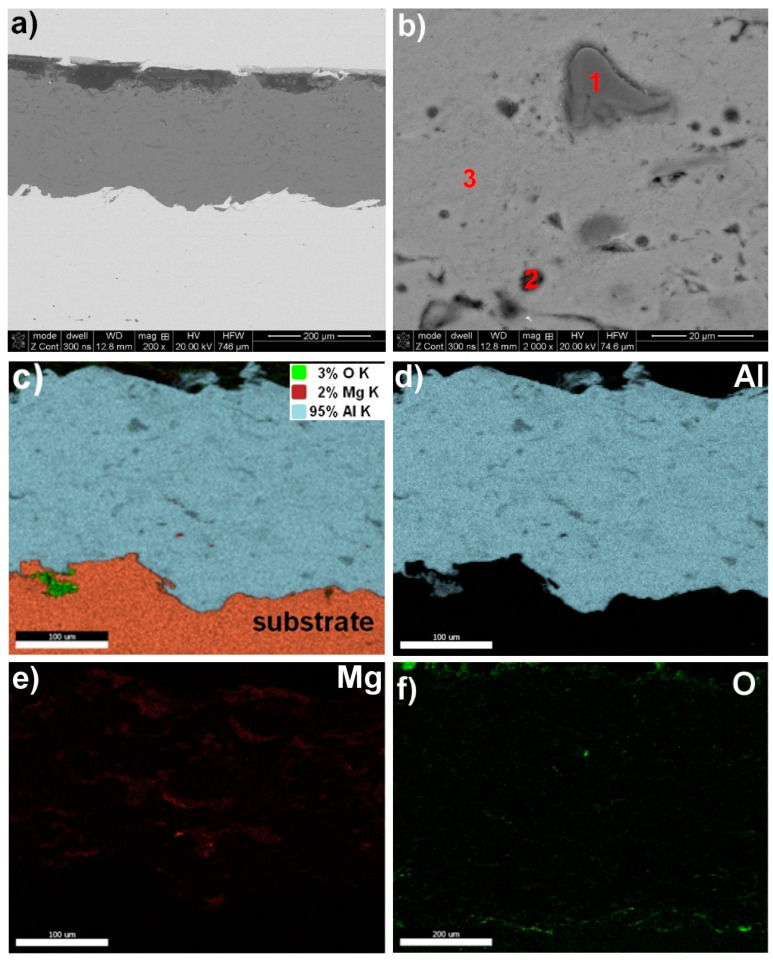
A typical microstructure of an Al5Mg arc–wire sprayed coating exhibiting type and grain morphology (**a**,**b**), with chemical composition based on SEM/EDS point (**b**) and map analysis (**c**–**f**).

A structural analysis of the coatings revealed that the arc spraying process conditions (Table 2) resulted in high-quality metallurgical properties of the protective coatings. These coatings had a thickness ranging from approximately 250 µm to 710 µm and a surface roughness within the Ra = (9.73–12.59) µm range (Figure 1, Figure 2, Figure 3 and Figure 4 and Table 5). The greater thickness of the aluminium coating (Table 5) was most likely due to the lower vapour pressure of aluminium compared to zinc, which evaporates during arc deposition due to its high vapour pressure [30]. It was found that the fabricated protective Zn, Al, Zn15Al, and Al5Mg coatings showed the structure, grain morphologies, and coating/substrate bonds typical for arc spraying, regardless of the sprayed materials, i.e., a microstructure comprising the crystallised, layered grains produced from the melted wire materials, which, in the arc process, become weakly oxidized, heavily impacting moulding and changing their grain geometry as they are converted into the coating. No structural discontinuities or microcracks were observed in the obtained SEM images.

**Figure 5:**
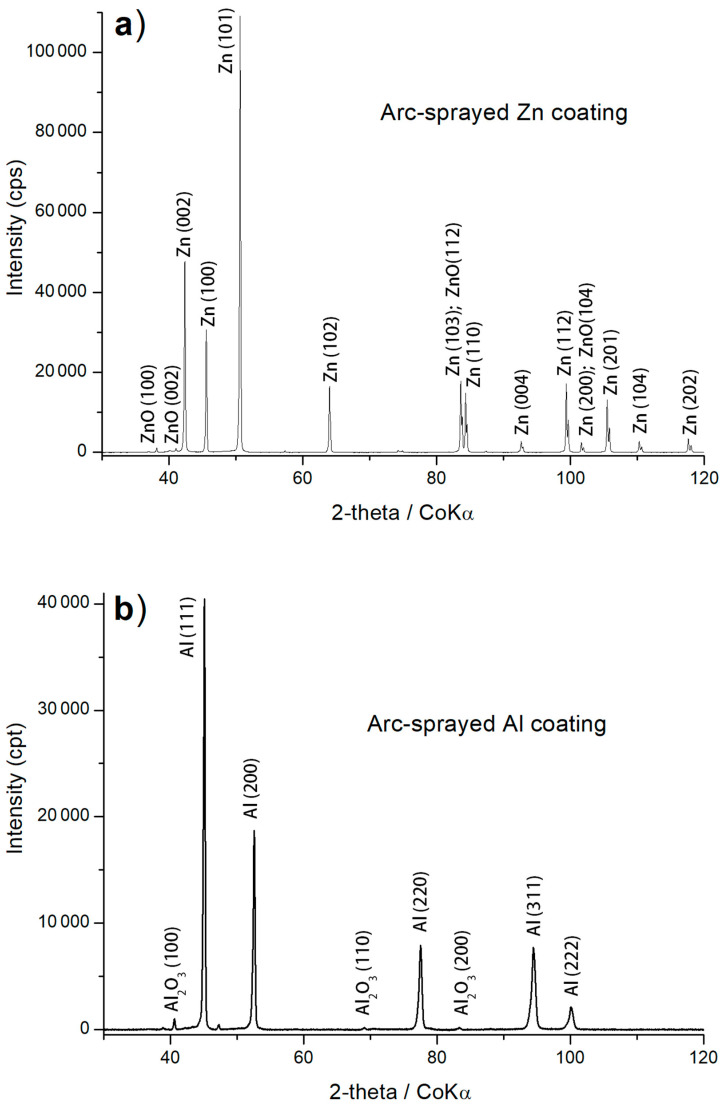

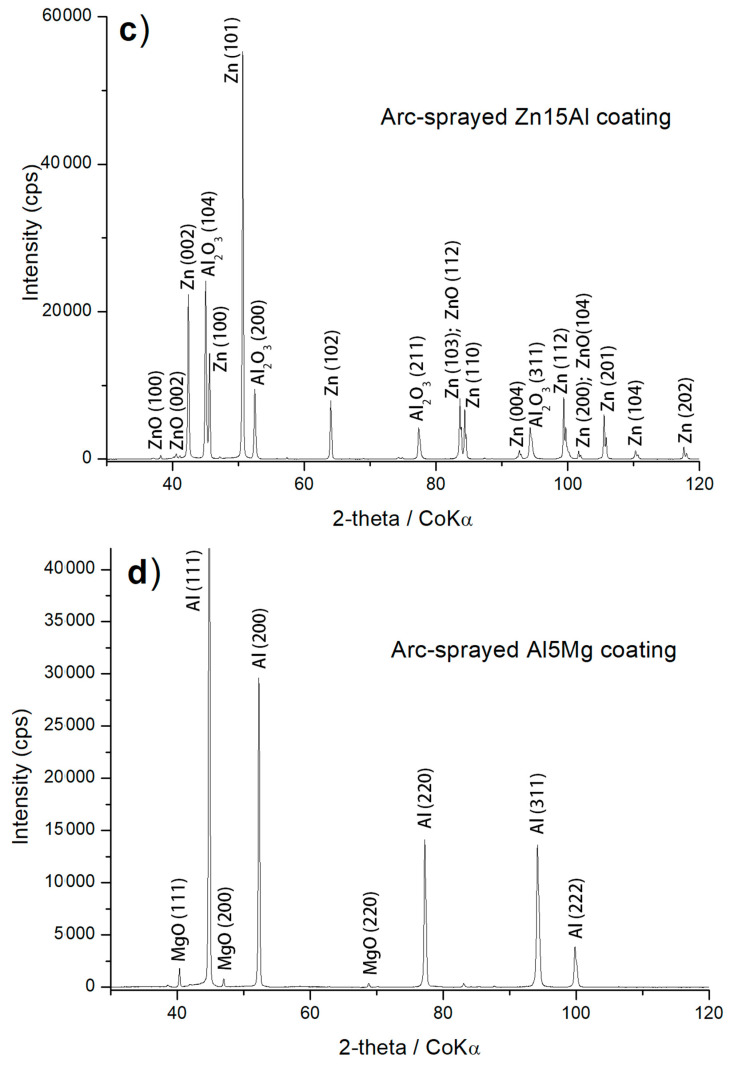
XRD patterns of the (**a**) Al, (**b**) Zn, (**c**) Zn15Al, and (**d**) Al5Mg arc-sprayed coatings [30].

The interface with the substrate material and the coating volume showed a maximum structure porosity of 2.5% for the aluminium coating, as shown in Figure 2 and Table 5. The porosities for the other coatings, i.e., Zn, Zn15Al, and Al5Mg, were below 0.4%. Upon examining the chemical compositions of the individual coatings, it was discovered through the SEM/EDS maps of the main alloying elements Zn, Al, Mg, and oxygen that molten wire particles carried in an air stream flowing at around 300 m/s experience oxidation. The average degree of oxidation for the Zn, Al, Zn15Al, and Al5Mg coatings was 4% wt., 2.5% wt., 2% wt., and 3% wt., respectively (Figure 1, Figure 2, Figure 3 and Figure 4 and Table 6). Oxygen micro-segregation was mainly observed in locations where oxide phases were formed, as identified via SEM/EDS and XRD tests (Figure 5).

Through XRD phase identification, it was determined that the arc-sprayed coatings composed of pure zinc and aluminium demonstrated a phase composition that was practically identical to that of the feedstock wire materials. However, these materials are chemically reactive and undergo oxidation during arc spraying [36,37]. In the coatings, oxides form, namely, ZnO and α-Al_2_O_3_ (Figure 5a,b), with a significant level of dispersion, as evidenced by the low intensity of the XRD reflections. The Zn15Al coating comprised grains with a lamellar structure, where the dominant phase was Zn (Figure 3). Additionally, the oxidation of molten particles led to the formation of oxide phases, notably α–Al_2_O_3_ oxide, as identified in XRD studies of the Zn15Al coating (Figure 5c).

A similar effect has been observed in Fe-Al type coatings during detonation gas spraying (DGS) [38,39]. As a result of the oxidation of the particles in the hot stream of gas-detonation products, a composite coating with a lamellar structure is formed on the basis of the Fe-Al phase with oxide participation, mainly α–Al_2_O_3_, which are created in situ in the DGS process at the grain boundaries [40,41,42]. No intermetallic phases are formed in the coating volume or at the interface with the steel substrate material in current arc-sprayed Al coatings.

In the Al5Mg coating, during arc spraying at temperatures above 3000 °C, MgO was also formed; this occurred as a dispersion of precipitates in the coating structure, where the matrix was a slightly oxidised α-phase as a solid solution of magnesium in aluminium with limited solubility (having the FCC {A1} lattice, just like aluminium); see Figure 5d. The SEM/EDS maps and point microanalysis (Figure 4) illustrate the distribution of the main alloying elements (Al and Mg) and oxygen in the cross-section of the Al5Mg coating. Table 6 presents the chemical compositions of the identified structural phases.

### 3.2. Electrochemical Analysis

Table 7 presents the results of our electrochemical LPR tests. The potentials for zinc coatings were lower in solutions with a lower pH. In such solutions, open circuit and corrosion potentials were measured as −1.04 ± 0.02 V and −1.06 ± 0.03 V, respectively. In contrast, solutions with higher salinity and lower pH values exhibited the most increased corrosion current density and corrosion rate values, measuring 3.525 ± 0.940 µA × cm^−2^ and 0.374. ± 100 g × m^−2^ × y^−1^, respectively. The aluminium coatings demonstrated a consistent potential value in the tested solutions, with corrosion and open circuit potentials measuring approximately −0.7 ± 0.05 V. The corrosion currents and rates were at their highest in high-salinity, low-pH solutions (0.271 ± 0.037 µA × cm^−2^, 29 ± 2.3 g × m^−2^ × y^−1^), while in low-salinity, high-pH solutions, they were considerably lower (0.069 ± 0.006 µA × cm^−2^, 2 ± 0.4 g × m^−2^ × y^−1^). The inclusion of aluminium in zinc coating results in a minor increase in open circuit and corrosion potentials. The values were highest for solutions with high salinity and high pH and for those with low salinity and low pH (−0.99 V for both open circuit and corrosion potentials). Low salinity and high pH solutions resulted in the lowest current density and wear (1.175 ± 0.017 µA × cm^−2^, 108.5 ± 1.6 g × m^−2^ × y^−1^), while solutions with high salinity and high pH exhibited the highest values of current and wear (1.175 ± 0.017 µA × cm^−2^, 108.5 ± 1.6 g × m^−2^ × y^−1^). Adding magnesium to aluminium coatings reduced corrosion and open circuit potentials in solutions with higher salinity and pH values (−0.72 V and −0.73 V, respectively). The current density and corrosion rate values typically increased, except for the values obtained with high salinity and low pH solutions (0.167 ± 0.007 µA × cm^−2^, 5.3 ± 0.2 g × m^−2^ × y^−1^). The open circuit and corrosion potentials of S235R steel were measured between −0.51 V and −0.41 V. All steel potentials were higher than those of the selected coatings, indicating a sacrificial corrosion protection mechanism. The average current density values ranged from 0.102 to 0.246 µA × cm^−2^. Higher values were observed in solutions with higher salinity and lower pH. These values were comparable to those obtained for aluminium materials and significantly lower than those for zinc-based materials.

**Figure 6:**
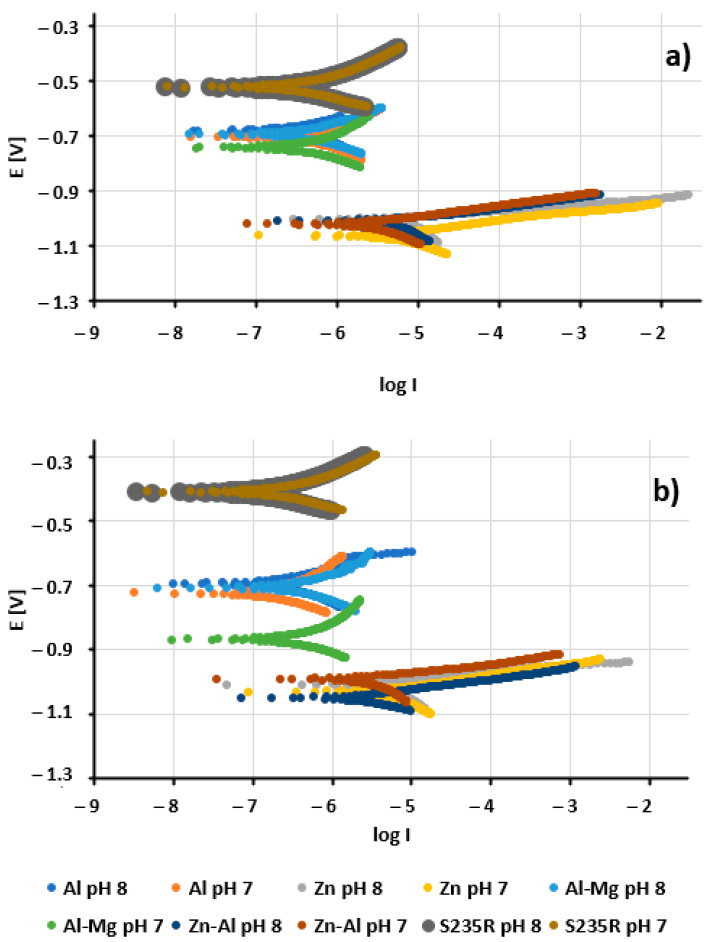
Tafel plots obtained in 30 g/L (**a**) and 7 g/L (**b**) solutions of salinity.

Averaged polarisation plots are exhibited in Figure 6. A general change in salinity from 7 g/L to 30 g/L did not result in substantial transformations in potentials; nevertheless, it affected the current density values and, consequently, the corrosion rate. Lower values of pH had a similar effect to an increase in salinity.

**Figure 7:**
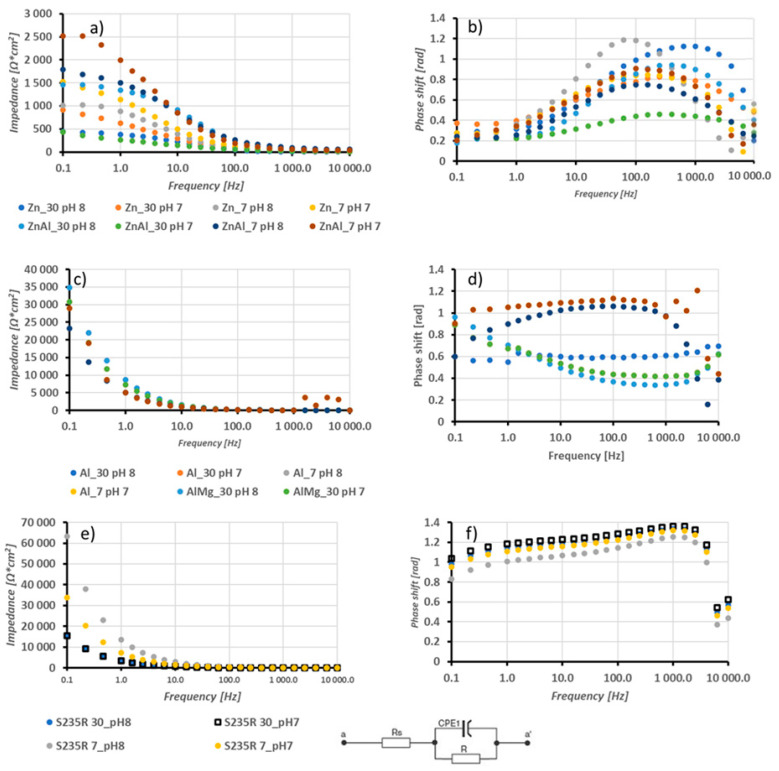
Bode plots for Zn and Zn15Al coatings (**a**,**b**), Al and Al5Mg coatings (**c**,**d**), and S235R steel (**e**,**f**), with corresponding equivalent circuits: (**a**,**c**,**e**) modulus–frequency; (**b**,**d**,**f**) phase–frequency.

Electrochemical impedance spectroscopy was carried out on the samples under investigation. Figure 7 presents the findings for all samples, including Bode plots for coatings dominant in zinc and aluminium and S235R steel itself. The circuit equivalent to these results is also shown. Resistance R_1_ is connected to the resistance of the coating due to porosity and oxide content, while R_2_ represents the charge transfer exchange of electrons [43,44].

The obtained resistance values of the coatings are presented in Table 8. The capacitance values of constant phase elements were not presented as, for all measured systems, their values fell within a range of 0 F × cm^2^. In the case of the zinc coatings, the lowest R_1_ was observed for solutions with low salinity and low pH (0.3 ± 0.1 Ω × cm^2^), while the highest was observed for low salinity and high pH (1.8 ± 0.1 Ω × cm^2^). Charge transfer resistance was highest in solutions of low salinity and low pH (201.3 ± 1.3 Ω × cm^2^) and lowest for high salinity and low pH (62.2 ± 0.8 Ω × cm^2^). Aluminium coatings demonstrated greater resistance when compared to zinc coatings. The coating with the lowest resistance was observed in high salinity and high pH solutions (23.5 ± 1.2 Ω × cm^2^), while the highest resistance was observed in low salinity and high pH solutions (59.9 ± 0.1 Ω × cm^2^). Charge transfer resistance was highest in solutions with low salinity and low pH (51,994 ± 13 Ω × cm^2^). The Zn15Al alloy coatings displayed greater resistance than pure zinc coatings and less resistance than aluminium coatings. The coating with the highest resistance was observed for low salinity and high pH values (40.1 ± 5.5 Ω × cm^2^), while the lowest resistance was found for high salinity and low pH (0.1 ± 0.02 Ω × cm^2^). Charge transfer resistance was highest for high salinity and high pH (555.3 ± 78.5 Ω × cm^2^) and lowest for low salinity and high pH (73.6 ± 12.5 Ω × cm^2^). The addition of magnesium to aluminium results in a reduction in coating resistance and an increase in charge transfer resistance. Solutions having lower salinity and pH (5.1 ± 0.05 Ω × cm^2^) indicated the lowest coating resistance, whereas those having higher salinity with low pH (32.7 ± 5.6 Ω × cm^2^) yielded the highest values. The charge transfer resistance appeared highest for solutions possessing low salinity and low pH (247,097 ± 345 Ω × cm^2^) and the lowest for solutions having low salinity with high pH (42,468 ± 258 Ω × cm^2^). Steel sample tests are better suited to different equivalent circuits than coating samples. This is because the possible lack of oxide layers and porous structures only makes it possible to observe resistance corresponding with charge transfer. The measured values ranged between 32,158 Ω × cm^2^ and 131,074 ± 547 Ω × cm^2^. The charge transfer resistance is directly proportional to the pH value and inversely proportional to salinity.

Table 9 demonstrates the impact of salinity and pH levels on the statistical results. The t-student coefficient values were calculated to determine the linear regression coefficients for electrochemical parameters, indicating significance (S) or non-significance (N) for salinity and pH. Insignificant values (N) are in italics.

According to the statistical analysis presented in Table 9, pH and salinity values substantially affect the corrosion rate, current density, and charge transfer resistance parameters. Salinity has almost no significant influence on potentials for aluminium and zinc-aluminium coatings. However, the potential values are influenced by pH for all coatings except aluminium coatings. Salinity does not affect the porous layer resistance except for those of Zn15Al coatings, while pH values control this parameter for mixed coatings. Corrosion current density and corrosion rate are parameters invariably influenced by both pH and salinity. Therefore, a regression coefficients analysis was performed and is available in Table 10. Positive coefficients indicate that the parameter increases with an increase in the factor value (pH and salinity). In contrast, negative coefficients indicate a decrease in the parameter value with an increase in the factor value.

**Figure 8:**
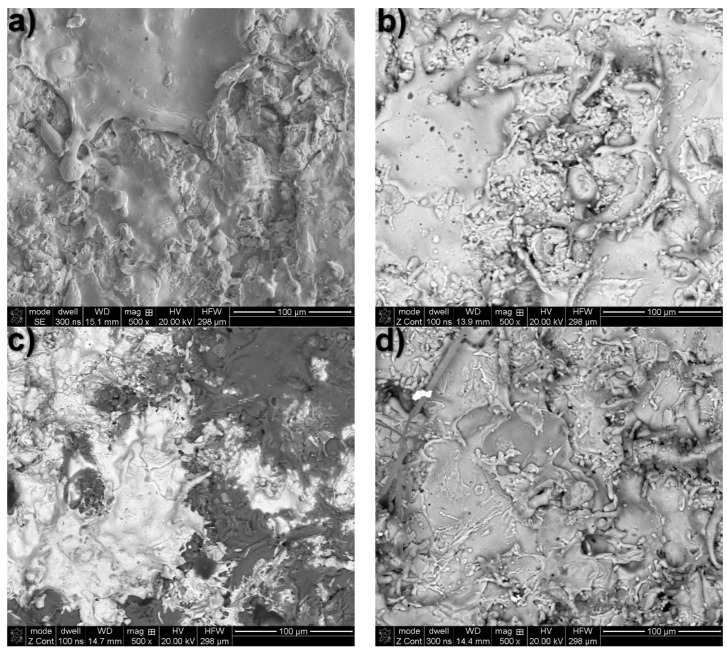
SEM/BSE images of corrosion products formed on arc-sprayed coatings after potentiodynamic testing in a 30 g/L saline solution: (**a**) Zn, (**b**) Al, (**c**) Zn15Al, (**d**) Al5Mg.

Following our potentiodynamic analysis of the coatings exposed to a solution with a salinity of 30 g/L (refer to Table 3), an SEM/EDS analysis was conducted again on the surface layer to determine the morphology and chemical composition of the corrosion products (Figure 8 and Table 11). The coatings’ surface layers displayed microstructural irregularities, including micropores formed by electrochemical etching in the grains in the electrolyte interaction zone. As a result, a relief structure formed with an irregular grain morphology, covered by a thin layer and forming coagulated corrosion products with trace elements of the working medium. The chemical composition of the corrosion products is shown in Table 11. The compact structure of the thin layer of the corrosion products which forms on the surface of the coatings is an important element in the sacrificial protection of S235R steel. It inhibits the penetration of aggressive chloride ions deep into the structure of the sprayed protective coatings. As Zn is more active than Al, it is likely that it dissolves preferentially in the Al15Zn coating, forming corrosion products with lower Zn content than the original arc-sprayed coating. This is supported by our SEM/EDS results (see Table 6 and Table 11 in correlation with Figure 8c). The electrochemical impedance spectroscopy (EIS) results show that the increase in charge transfer resistance of the Al5Mg coating was affected by the proportion of Mg, which significantly impacted the corrosion resistance of the arc-sprayed Al coating. This was also confirmed by Han-Seung Lee et al., who showed that an Al-Mg coating provides more than 6.5 times longer sacrificial protection of a steel substrate compared to an Al-Zn coating, i.e., 792 h and 120 h exposure, respectively, to an industrial environment solution containing aggressive chloride and carbonate ions [5].

In summary, the results lead to the conclusion that all investigated coatings provide sacrificial protection to S235R steel. Generally, higher salinity levels lead to an increase in the corrosion rate of most metals [45]. This was confirmed by the obtained results, which showed that as salinity increases, the corrosion rates of aluminium, zinc, and zinc-aluminium coatings also increase. In contrast, for the Al5Mg coating, the corrosion rate decreased with increasing salinity and decreasing pH. Other studies [7,46] have also observed unusual behaviour of electrochemical properties caused by the addition of magnesium. This may result from a high concentration of carbonate anions that promote the production of protective carbonate layers [47]. However, at significantly higher pH levels, the formation of larger quantities of Mg(OH)_2_ occurs, leading to increased corrosion in Al-Mg alloys [48,49,50]. It should be also noted that magnesium as a pure metal is not resistant under low salinity conditions or at high pH [51]. Research conducted by other authors [52] has shown that when magnesium is in the matrix of less reactive metals, it is the main element to be depleted. In contrast, aluminium shows high stability in the pH range of 4–9 [53]; its primary corrosion products are aluminium oxides and hydroxides.

In the case of zinc-aluminium coatings, corrosion resistance increases with decreasing pH. The formation of aluminium hydroxides causes this, which is the primary corrosion mechanism for Zn-Al coatings [54,55]. This process occurs in two stages: first, the formation of hydroxides, and second, the synthesis of non-soluble salts. Carbonates are particularly useful for increasing corrosion protection among those substances [55]. Other research has noted that the formation of layered double hydroxides was observed [16]. These inorganic substances have complex structures containing zinc, aluminium, hydroxy groups, and carbonate groups. These structures are created in more alkaline conditions. In more acidic conditions, layered double hydroxides are probably present in lower amounts due to a lower amount of hydroxy anions and the production of carbon dioxide from carbonates. A similar situation occurs for zinc coatings. Corrosion products consist mainly of hydroxides and mixed hydroxy salts containing chlorides and carbonates [56].

## 4. Conclusions

The paper describes the corrosion resistance of four different arc-sprayed coatings (Zn, Al, Zn15Al, and Al5Mg) in relation to the salinity and acidity of seawater in light of global climate change due to greenhouse gas emissions.

Essential research conclusions are the claim that the applied parameters of arc spraying allow for a dense structure of the coatings with low porosity (from 0.3% to 2.5%) with the participation of oxide phases, i.e., ZnO, α–Al_2_O_3_, and MgO, formed in the structure of the coatings. The corrosion rate was highest in solutions with high salinity and low pH. Aluminium coatings exhibited greater corrosion resistance in the tested solutions than zinc coatings. Zn15Al alloy coatings demonstrated superior corrosion resistance compared to Zn coatings but inferior corrosion resistance to aluminium coatings. Furthermore, these coatings experienced a boost in their corrosion resistance as the pH level increased. Adding Mg 5% wt.% to aluminium reduced the corrosion potential in higher salinity and pH solutions. The corrosion rate of Zn, Al, and Zn15Al coatings increased with increasing salinity, while the corrosion rate of the Al5Mg coating decreased as salinity and pH decreased. Hydroxy zinc carbonates are the most likely product of corrosion in Zn15Al coatings, particularly in seawater with high CO_2_ saturation. Al5Mg coatings, on the other hand, offered excellent protection in acidic environments due to the sacrificial role of magnesium. Both sacrificial corrosion mechanisms provided better protection in more acidic environments.

Based on this research analysis, it can be concluded that Zn15Al and Al5Mg alloy coatings are advantageous for seawater applications.

## Figures and Tables

**Table 1:** Chemical composition of S235JR steel plate [30].

Element	C	P	N	S	Fe
wt. %	0.18	0.013	0.009	0.027	rem.

**Table 2:** Arc-spraying parameters applied to obtain four types of coatings from 2 mm feedstock wire materials.

Arc Spraying Parameters	Feedstock Wire Materials Used in Thermal Arc-Spraying Experiments			
	Zn	Al	Zn15Al	Al5Mg
Atomising gas pressure [MPa]	0.65	0.65	0.65	0.65
Arc current [A]	50	50	100	50
Arc voltage [V]	20	36	25	36
Power input [kW]	1	1.8	2.5	1.8
Distance spraying [mm]	150–250 (for all types of coatings)			

**Table 3:** Composition of experimental solutions used for electrochemical testing.

Compound	Mass of Compound per Litre of Solution [g] (Salinity 30 g/L)	Mass of Compound per Litre of Solution [g] (Salinity 7 g/L)
NaCl	20.5	4.78
MgCl_2_	4.3	1
Na_2_SO_4_	3.4	0.79
CaCl_2_	1.0	0.23
KCl	0.6	0.14
NaHCO_3_	0.2	0.05

**Table 4:** Experimental plan for electrochemical testing.

Solution Number	Salinity (g/L)	pH
1	30	8
2	30	7
3	7	8
4	7	7

**Table 5:** Properties of the obtained arc-sprayed coatings.

Arc–Wire Sprayed Coatings	Thickness (μm)	Porosity (%)	Roughness R_a_ (µm)	Phase Composition (XRD Patterns)
Zn	430 ± 23	0.3 ± 0.1	9.73	zinc, zinc oxides
Al	710 ± 19	2.5 ± 0.2	12.20	aluminium, α–Al_2_O_3_
Zn15Al	592 ± 32	0.35 ± 0.1	12.59	zinc, ZnO, α–Al_2_O_3_
Al5Mg	250 ± 19	0.4 ± 0.15	12.06	aluminium {Mg(Al) α–phase}, MgO

**Table 6:** Semiquantitative EDS analysis (at.%) of arc wire sprayed with four types of coatings (Zn, Al, Zn15Al, and Al5Mg).

Designation of Grain Area according to Figure 1, Figure 2, Figure 3 and Figure 4	Zn	Al	Mg	O
Zn coating—Figure 1				
mapping	~96.0	−	−	~4.0
Al coating—Figure 2				
mapping	−	~97.5	−	~2.5
Zn15Al coating—Figure 3				
mapping	56.0	42.0	−	~2.0
Al5Mg—Figure 4b				
1—grey	−	68.8	2.0	~29.2
2—dark grey	−	94.3	1.5	~4.2
3—light grey	−	96.7	1.0	~2.3
mapping	−	95.0	2.0	~3.0

**Table 7:** Electrochemical parameters obtained from LPR tests.

Zn				
Solution No	E_ocp_ (V)	E_corr_ (V)	I_corr_ (µA × cm^−2^)	r (g × m^−2^ × y^−1^)
1	–1 ± 0.01	–1 ± 0.01	3.149 ± 0.923	334.5 ± 98
2	–1.04 ± 0.02	–1.06 ± 0.03	3.525 ± 0.940	374.5 ± 100
3	–1 ± 0.01	–1.01 ± 0.01	2.068 ± 0.731	220 ± 78
4	–1.02 ± 0.02	–1.03 ± 0.02	3.048 ± 0.701	324 ± 75
**Al**				
**Solution No**	**E_ocp_ (V)**	**E_corr_ (V)**	**I_corr_ (µA × cm^−2^)**	**r (g × m^−2^ × y^−1^)**
1	–0.7 ± 0.02	–0.68 ± 0.04	0.186 ± 0.028	5 ± 0.8
2	–0.69 ± 0.02	–0.7 ± 0.03	0.271 ± 0.037	29 ± 2.3
3	–0.69 ± 0.04	–0.7 ± 0.07	0.069 ± 0.006	2 ± 0.4
4	–0.7 ± 0.04	–0.73 ± 0.06	0.093 ± 0.020	2.73 ± 2
**Zn15Al wt.%**				
**Solution no**	**E_ocp_ (V)**	**E_corr_ (V)**	**I_corr_ (µA × cm^−2^)**	**r (g × m^−2^ × y^−1^)**
1	–0.99 ± 0.01	–0.99 ± 0.02	2.410 ± 0.125	211 ± 11
2	–1.01 ± 0.01	–1.02 ± 0.04	1.919 ± 0.041	227 ± 1.2
3	–1.02 ± 0.01	–1.05 ± 0.05	1.175 ± 0.17	108.5 ± 1.6
4	–0.99 ± 0.01	–0.99 ± 0.03	1.430 ± 0.37	125.6 ± 3.2
**Al5Mg wt.%**				
**Solution no**	**E_ocp_(V)**	**E_corr_ (V)**	**I_corr_ (µA × cm^−2^)**	**r (g × m^−2^ × y^−1^)**
1	–0.72 ± 0.01	–0.73 ± 0.01	0.209 ± 0.013	6.1 ± 0.4
2	–0.68 ± 0.07	–0.66 ± 0.07	0.168 ± 0.007	5.3 ± 0.2
3	–0.69 ± 0.02	–0.7 ± 0.01	0.155 ± 0.011	5.1 ± 0.3
4	–0.7 ± 0.01	–0.7 ± 0.03	0.178 ± 0.016	5.2 ± 0.5
**S235R**				
**Solution no**	**E_ocp_(V)**	**E_corr_ (V)**	**I_corr_ (µA × cm^−2^)**	**r (g × m^−2^ × y^−1^)**
1	−0.51 ± 0.01	−0.52 ± 0.01	0.225 ± 0.013	13.76 ± 0.81
2	−0.52 ± 0.02	−0.52 ± 0.01	0.246 ± 0.014	15.02 ± 0.89
3	−0.40 ± 0.02	−0.41 ± 0.02	0.102 ± 0.006	6.25 ± 0.37
4	−0.41 ± 0.02	−0.41 ± 0.02	0.143 ± 0.008	8.76 ± 0.52

**Table 8:** Equivalent circuit resistance for the investigated solutions.

Zn		
Solution No.	R_1_ [Ω × cm^2^]	R_2_ [Ω × cm^2^]
1	1.5 ± 0.1	167.4 ± 27.9
2	1.3 ± 0.2	62.2 ± 0.8
3	1.8 ± 0.1	119.0 ± 5.7
4	0.3 ± 0.1	201.3 ± 1.3
**Al**		
**Solution no.**	**R_1_ [Ω × cm^2^]**	**R_2_ [Ω × cm^2^]**
1	23.5 ± 1.2	31,140 ± 4
2	36.3 ± 0.2	21,820 ± 0.8
3	59.9 ± 0.1	35,714 ± 5.7
4	29 ± 0.1	51,994 ± 13
**Zn15Al**		
**Solution no.**	**R_1_ [Ω × cm^2^]**	**R_2_ [Ω × cm^2^]**
1	4.1 ± 0.5	555.3 ± 78.5
2	0.1 ± 0.02	251.3 ± 16
3	40.1 ± 5.5	73.6 ± 12.5
4	3.7 ± 0.6	229.7 ± 7.3
	**Al5Mg**	
**Solution no.**	**R_1_ [Ω × cm^2^]**	**R_2_ [Ω × cm^2^]**
1	7.4 ± 1.3	71,775 ± 472
2	32.7 ± 5.6	83,236 ± 135
3	5.1 ± 0.05	42,468 ± 258
4	25.9 ± 4.4	247,097 ± 345
**S235R**		
**Solution no.**	**R_1_ [Ω × cm^2^]**	**R_2_ [Ω × cm^2^]**
1	0	32,602 ± 136
2	0	32,158 ± 134
3	0	131,074 ± 547
4	0	69,980 ± 292

**Table 9:** T-student’s coefficient values for the relevance of salinity and pH.

Salinity	Electrochemical Parameter					
Coating	E_stat_	E_corr_	I_corr_	r	R_1_	R_2_
Al	*0.0 N*	*1.9 N*	158.9 S	50.3 S	0.8 S	92.6 S
Zn	3.2 S	3.0 S	776.7 S	253.3 S	0.5 S	5.5 S
Zn15Al	*1.8 N*	*2.2 N*	897.0 S	266.2 S	*3.5 N*	12.9 S
Al5Mg	5.7 S	6.9 S	131.4 S	25.2 S	*2.1 N*	207.0 S
**pH**	**Electrochemical parameter**					
**Coating**	**E_stat_**	**E_corr_**	**I_corr_**	**r**	**R_1_**	**R_2_**
Al	*1.1 N*	*2.1 N*	96.7 S	45.5 S	*1.0 N*	92.6 S
Zn	4.6 S	5.5 S	724.7 S	236.0 S	*0.7 N*	*2.8 N*
Zn15Al	3.6 S	3.6 S	637.2 S	203.2 S	3.5 S	7.1 S
Al5Mg	5.2 S	4.9 S	80.5 S	18.1 S	4.1 S	223.1 S

**Table 10:** Regression coefficients for corrosion current density and corrosion rate.

Salinity		
Coating	I_corr_	r
Al	73.83	7.40
Zn	389.43	41.41
Al5Mg	–17.07	–0.63
Zn15Al	421.71	37.15
**pH**		
**Coating**	**I_corr_**	**r**
Al	–27.34	–3.04
Zn	–339.23	–35.99
Al5Mg	6.40	0.32
Zn15Al	212.68	21.67

**Table 11:** SEM/EDS microanalysis of the chemical composition on the surface of coatings (Zn, Al, Zn15Al, and Al5Mg) after potentiodynamic tests in a 30 g/L saline solution.

Analysis Area according to Figure 8a–d	Content, at.%						
	Zn	Al	Mg	O	Cl	K	Ca
Zn coating—Figure 8a							
mapping	~94.7	−	−	~4.9	~0.18	~0.22	−
Al coating—Figure 8b							
mapping	−	~89.4	−	~9.7	~0.33	~0.14	~0.43
Zn15Al coating—Figure 8c							
mapping	~35	~55	−	~9	~0.37	~0.12	~0.5
Al5Mg—Figure 8d							
mapping	−	~92.0	~1.8	~5.6	~0.54	~0.06	~0.32

## Data Availability

Data is available on request from authors.
